# Investigation on Applicability and Limitation of Cosine Similarity-Based Structural Condition Monitoring for Gageocho Offshore Structure

**DOI:** 10.3390/s22020663

**Published:** 2022-01-15

**Authors:** Byungmo Kim, Jaewon Oh, Cheonhong Min

**Affiliations:** 1 Department of Ocean Engineering, Korea Maritime and Ocean University, Busan 49112, Korea; bmkim@g.kmou.ac.kr; 2 Offshore Industries R&BD Center, Korea Research Institute of Ships & Ocean Engineering (KRISO), Geoje 53201, Korea; herotaker@kriso.re.kr

**Keywords:** damage detection, cosine similarity, structural health monitoring, Gageocho Ocean Research Station, structural integrity assessment

## Abstract

The key to coping with global warming is reconstructing energy governance from carbon-based to sustainable resources. Offshore energy sources, such as offshore wind turbines, are promising alternatives. However, the abnormal climate is a potential threat to the safety of offshore structures because construction guidelines cannot embrace climate outliers. A cosine similarity-based maintenance strategy may be a possible solution for managing and mitigating these risks. However, a study reporting its application to an actual field structure has not yet been reported. Thus, as an initial study, this study investigated whether the technique is applicable or whether it has limitations in the real field using an actual example, the Gageocho Ocean Research Station. Consequently, it was found that damage can only be detected correctly if the damage states are very similar to the comparison target database. Therefore, the high accuracy of natural frequencies, including environmental effects, should be ensured. Specifically, damage scenarios must be carefully designed, and an alternative is to devise more efficient techniques that can compensate for the present procedure.

## 1. Introduction

### 1.1. Background

At present, extreme climate change has caused many disasters worldwide. For instance, a German meteorologist stated that the level of flood severity in Germany in July 2021 was on a level not seen for at least 500 or even 1000 years, and a spokesman for the German Weather Service also said that such an event had probably not occurred in a millennium [1]. Around the same time, there was a record of heavy rain in a central China province, and many Chinese meteorologists considered that the downpour may have been one in a thousand years [2]. Numerous casualties have occurred as a result of these disasters. In addition, various other types of catastrophes, such as extensive forest fires, severe heat waves or cold waves, and hurricanes, have increased worldwide.

Global warming is considered to be the root cause of extreme weather events that have become more diverse, sudden, and powerful. Recently, the Intergovernmental Panel on Climate Change (IPCC) of the United Nations (UN) approved the sixth assessment report [3], which stated that within the next two decades, global warming is expected to exceed the temperature increase limit of 1.5 °C decided as per the Paris Climate Agreement in 2015. This implies the cause of faster than expected climate change events that have been occurring. In addition, it mentioned that the Earth’s temperature increment exceeding the target of 1.5 °C would result in significantly increased catastrophic consequences in all aspects compared to the present situation. Therefore, immediate, extreme high-intensity innovation has been recommended in an effort to sharply reduce the emission of greenhouse gases to carbon neutrality, and subsequently, to absorb the carbon discharged into the atmosphere to achieve the target.

The priority is to change energy governance from carbon-based to green energy, and many nations have already begun exerting efforts to achieve this. Specifically, at present, the focus has been on ocean renewable energy resources such as offshore wind turbines, and the recent IPCC report has stated such a trend is a correct approach that can be used to tackle the issue. This is because relevant technologies are already sufficiently mature to be commercialized compared to other renewable energy sources, such as hydrogen- or ammonia-based energy and nuclear fusion energy. Thus, extensive utilization is possible in less time. This finding is also supported by the energy market trend. For example, the Levelized cost of electricity (LCOE) of newly added renewable energy plants in 2020 is cheaper than that of existing coal-fired plants. In the case of offshore wind turbines, the LCOE has continuously decreased by approximately 48% over the last decade [4], which indicates that this approach adheres well to the urgency highlighted in the report. Therefore, it is necessary to rapidly construct and operate a large amount of offshore energy power generation systems that can aid in replacing power generation using fossil fuels. Jeremy Rifkin, a leading futurist, said “The Earth only has a razor blade-thin amount of time left” [5].

The rapid construction of many new offshore energy generators should also be accompanied by stable operational capabilities. This is important because of the increase in the inherent risk in the offshore energy systems than before global warming because the history of modern marine statistics is short and meteorological and oceanographic factors have gradually changed. Thus, current design standards based on climate history cannot guarantee safety in case of environmental outliers triggered by rapid climate change in the future. Consider following the reasoning: offshore renewable energy resources, such as wind, waves, and currents, are the medium of global energy circulation on Earth and are directly influenced by global warming. At the same time, they act directly on offshore structures as external forces. Specifically, the increase in the sea surface temperature can cause the maximum wind speed of a hurricane to increase by 5% per 1 °C in theory [6], thereby also magnifying the wind load by approximately 10%. As a practical example, the Gageocho Ocean Research Station (ORS), a jacket-type offshore structure, was damaged by typhoons that were much stronger than expected than those that were anticipated during the design process. However, due to the design standards having been established with a wide agreement based on accumulated research for a long time, immediately changing and applying them to offshore structures that have been built is challenging. Therefore, it is better to manage this issue during the lifetime of structures at the maintenance stage during their operation.

### 1.2. Limitation of Traditional Maintenance Strategy and Research Purpose

**Figure 1:**
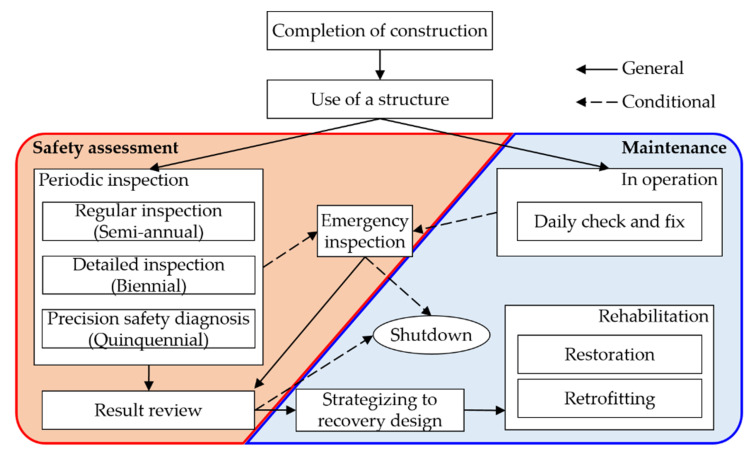
Flowchart of the conventional safety assessment and maintenance processes.

The conventional safety assessment and maintenance processes of a structure or building are enforced under relevant rules or regulations. The procedure prescribed by [7] in South Korea is comprehensively illustrated in Figure 1, which was referred to in [8] and [9]. Specifically, the flow for the review and assessment of safety and the task for operational maintenance are separated from each other. For structural integrity identification, the process begins when specialists visit the site. Consequently, inspection, diagnosis, and preventive maintenance are performed if there is no evidence of damage. Otherwise, inspection, damage recognition, diagnosis, cause identification, countermeasure establishment, and rehabilitation must be conducted. In addition, the process is performed periodically, but not at all times, during operation.

**Figure 2:**
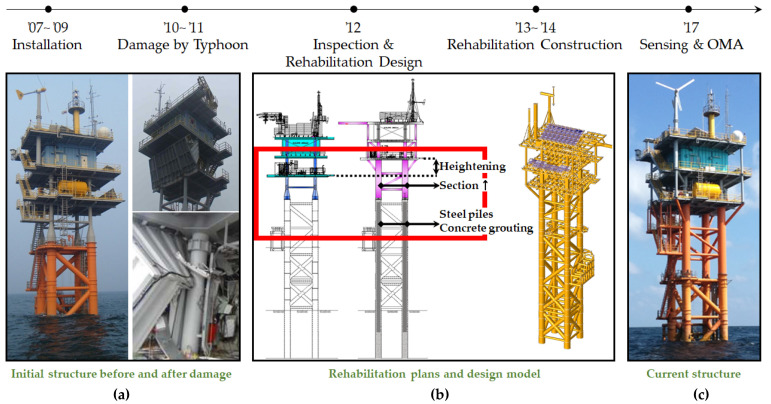
Installation, damage, and rehabilitation history of Gageocho ORS. (**a**) Initial structure before and after damage [13] (**b**) Rehabilitation plans [12] and design model [13] (**c**) Current structure [14].

However, there are certain disadvantages in its application to offshore structures. First, safety assessment is different from maintenance; thus, inspection experts and diagnosis experts are needed along with operation workers. Moreover, experts do not reside within the structure because the tasks are periodic. In addition, inspection and diagnosis are performed separately. Further, the critical environmental forces are accidental, and sites that are far from land have poor approachability; therefore, timeliness is limited. Therefore, offshore structures are difficult to manage from a preventive perspective. This is the reason that the traditional process almost always begins after the occurrence of critical damage and also accounts for the high financial and time costs. For example, approximately two years were required to administer the removal and replacement of the nacelle and blades of a wind turbine, which dropped into the sea due to a mechanical failure in the Samsø offshore wind farm [10,11]. In addition, approximately four years were required to reconstruct the Gageocho ORS after damage [12], as shown in Figure 2.

To solve this disadvantage, a variety of studies emphasizing structural health monitoring (SHM) have been conducted, and recent research trends have involved new approaches, such as artificial intelligence and big data, in the face of the 4th Industrial Revolution. In particular, data-driven SHM is considered to be promising and has garnered attention. This is because it is independent of a computational model and is thus better matched to the concept of real-time SHM [15]. As a representative study, the SHM procedure was established from the perspective of big data [16], and a research case applied a complete data-driven SHM to bridges [17]. Although these studies indicate steady growth, there is still room for development to realize perfect utilization in the field of offshore structures [18]. The reason for this is that the appropriate number of sensors required for the accurate computation of the modal parameters for SHM [19] is important, while the size of an offshore structure is large and the degree of structural complexity is high, and the number of sensors needed depends on those structural characteristics. Certain studies have concentrated on making data-driven SHM work effectively [20,21,22], while others have proposed diverse hybrid methods [23,24,25].

This study focused on exteriorizing the overall outline of the requirements for performing efficient safety assessments and maintenance. First, the most important factor is timeliness. This can be achieved through real-time inspection instead of periodic inspection. Second, inspection and diagnosis should be conducted in conjunction; that is, the sensing and diagnosis should not be manually conducted by different experts but should rather be automated. Third, the diagnosis should be interpretable to maintenance workers who are not structural safety specialists. Consequently, to develop such a converged SHM technique, a cosine similarity-based damage identification method that is capable of detecting single damage was proposed [26]. Kim et al. [27] extended this idea to determine multiple damages, and as an example, a simple portal frame was used to validate the performance. 

The process is briefly explained as follows: After composing many damage scenarios for a structure and using the finite element (FE) model to obtain the natural frequency change rate for each damage scenario in advance, they were compared to the actual natural frequency change rate in real-time by considering the cosine similarity. Consequently, the most similar damage scenarios based on similarity ranking are listed. The merits of this are as follows: first, the information for various damage states is preliminarily set using the FE model in the first step; second, the cosine similarity is very simple and can be quickly calculated, and thus, the inspection and diagnosis can be directly connected. Finally, maintenance workers without professional knowledge can intuitively understand the current state of the structure. Thus, this technique adheres to three previously stated requirements. In maintenance during operation, only measured natural frequencies are required, and no simulation model or difficult theories and techniques are required [26,27]. Therefore, it can be considered to be a hybrid and model-free SHM method. However, this has not been tested for actual offshore structures. Therefore, as a follow-up study, in this study, the technique was applied to an actual offshore jacket-type structure, the Gageocho ORS, to verify and discuss its capability and limitations.

## 2. Methodology

### 2.1. Cosine Similarity-Based SHM

**Figure 3:**
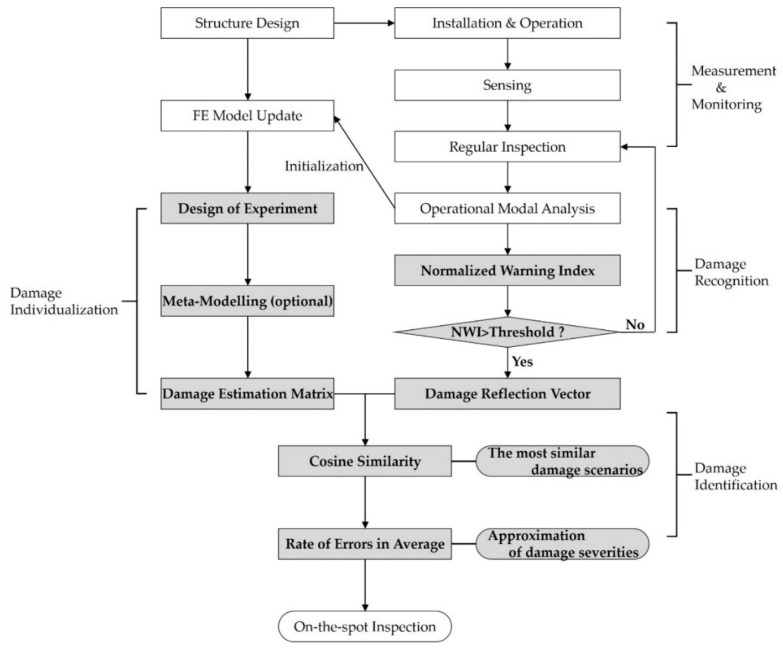
Entire flowchart of the cosine similarity-based SHM [27].

As shown in Figure 3 and referring to the two previous studies [26,27], the SHM process is composed of three sub-processes: the individualization, recognition, and identification of damage, which are shaded in the figure. A detailed explanation of the process and the parameters can be found in [27]; therefore, they are only briefly introduced in this study.

Regarding damage individualization, the damage estimation vector (DEV) is the rate of change in the natural frequencies before and after the damage of a pre-updated FE model, as shown in Equations (1) and (2). Further, as seen in Equation (3), the damage estimation matrix (DEM) is a matrix consisting of the DEVs and the target of the similarity comparison.

(1) $$z_{i,j}=\frac{f_{i,j}^{*}-f_{j}}{f_{j}},$$

(2) $$s_{i}={\left|\begin{array}{cc} \begin{array}{ccc} z_{i,1} & z_{i,2} & \cdots \end{array} & \begin{array}{ccc} z_{i,j} & \cdots & z_{i,n} \end{array} \end{array}\right|}^{T},$$

(3) $$S={\left|\begin{array}{cc} \begin{array}{cc} s_{1} & s_{2} \end{array} & \begin{array}{cc} \cdots & s_{m} \end{array} \end{array}\right|}^{T}=\left[\begin{array}{cc} \begin{array}{cc} z_{1,1} & z_{1,2}\\ z_{2,1} & z_{2,2} \end{array} & \begin{array}{cc} \cdots & z_{1,n}\\ \cdots & z_{2,n} \end{array}\\ \begin{array}{cc} \vdots & \vdots \\ z_{m,1} & z_{m,2} \end{array} & \begin{array}{cc} z_{i,j} & \vdots \\ \cdots & z_{m,n} \end{array} \end{array}\right],$$

where *i* and *m* are the arbitrary index and total numbers of the designed damage conditions, respectively, *j* and *n* are arbitrary and maximum available mode numbers, respectively, *f_j_* is the *j*th natural frequency of the undamaged FE model, $f_{i,j}^{*}$ is the *j*th natural frequency of the FE model in the *i*th damage case, *z_i,j_* is the rate of change of the *j*th natural frequency under the *i*th damage condition, ***s_i_*** is the DEV for the *i*th damage scenario, and ***S*** is the DEM for all of the designed damage conditions. 

In terms of damage recognition, among the actual natural frequencies cumulated as Equation (4), the rate of change in the natural frequencies between two sequential data points is calculated using Equation (5). This is known as the warning index (WI). Further, it is normalized by its mean and standard deviation, which is the normalized warning index (NWI), as expressed in Equation (6). For NWI values larger than a threshold, the occurrence of damage is perceived, and subsequently, the damage reflection vector (DRV) and the rates of changes in natural frequencies at that time are calculated using Equation (7).

(4) $$D={\left|\begin{array}{cc} \begin{array}{ccc} d_{1} & d_{2} & \cdots \end{array} & \begin{array}{ccc} d_{k} & \cdots & d_{p} \end{array} \end{array}\right|}^{T}=\left[\begin{array}{cc} \begin{array}{cc} f_{1}^{1} & f_{2}^{1}\\ f_{1}^{2} & f_{2}^{2} \end{array} & \begin{array}{cc} \cdots & f_{n}^{1}\\ \cdots & f_{n}^{2} \end{array}\\ \begin{array}{cc} \vdots & \vdots \\ f_{1}^{p} & f_{2}^{p} \end{array} & \begin{array}{cc} f_{j}^{k} & \vdots \\ \cdots & f_{n}^{p} \end{array} \end{array}\right],$$

(5) $$WI_{j}^{p}=\frac{f_{j}^{p}-f_{j}^{p-1}}{f_{j}^{p-1}},$$

(6) $$NWI_{j}^{p}=\frac{WI_{j}^{p}-\mu_{j}^{p-1}}{\sigma_{j}^{p-1}},$$

(7) $$h={\left|\begin{array}{cc} \begin{array}{cc} WI_{1}^{d} & WI_{2}^{d} \end{array} & \begin{array}{cc} \cdots & WI_{n}^{d} \end{array} \end{array}\right|}^{T}={\left|\begin{array}{cc} \begin{array}{cc} \frac{f_{1}^{d}-f_{1}^{d-1}}{f_{1}^{d-1}} & \frac{f_{2}^{d}-f_{2}^{d-1}}{f_{2}^{d-1}} \end{array} & \begin{array}{cc} \cdots & \frac{f_{n}^{d}-f_{n}^{d-1}}{f_{n}^{d-1}} \end{array} \end{array}\right|}^{T},$$

where *k* and *p* are the arbitrary and the most recent cycle numbers of the inspections, respectively, *f_j_^k^* is the *j*th natural frequency at the *k*th inspection cycle, ***d_k_*** is the vector of the natural frequencies at the *k*th inspection cycle, ***D*** is a matrix consisting of all of the natural frequencies obtained following the most recent inspection, *WI_j_^p^* is the warning index of the *j*th natural frequency at the *p*th inspection cycle, *μ_j_^p−1^* is the mean of the warning indices, that is, the average change rate of the *j*th natural frequency for the *j − 1st* natural frequency by the *p-1st* inspection cycle, *σ_j_^p−1^* is the standard deviation of the warning indices by the *p-1st* inspection cycle, *d* is the inspection cycle number when damage is recognized, and ***h*** is the DRV at the time.

Finally, the cosine similarity between DEM and DRV was estimated using Equation (8). For a more precise diagnosis, the rate of errors in detection (RED) can be calculated using Equation (9), whereas the average of its element, the rate of errors on average (REA), as expressed in Equation (10), is used to interpolate or extrapolate the reduced value of Young’s modulus.

(8) $$CS_{i}=\frac{s_{i}\cdot h}{\left\|s_{i}\|\|h\right\|}=\frac{\sum_{j=1}^{n}z_{i,j}WI_{j}^{d}}{\sqrt{\sum_{j=1}^{n}{\left(z_{i,j}\right)}^{2}}\sqrt{\sum_{j=1}^{n}{\left(WI_{j}^{d}\right)}^{2}}},$$

(9) $$g_{i}=\frac{s_{i}-h}{h}={\left|\begin{array}{cc} \begin{array}{ccc} \frac{z_{i,1}-WI_{1}^{d}}{WI_{1}^{d}} & \frac{z_{i,2}-WI_{2}^{d}}{WI_{2}^{d}} & \cdots \end{array} & \begin{array}{ccc} \frac{z_{i,j}-WI_{j}^{d}}{WI_{j}^{d}} & \cdots & \frac{z_{i,n}-WI_{n}^{d}}{WI_{n}^{d}} \end{array} \end{array}\right|}^{T},$$

(10) $$r_{i}=\frac{1}{n}\sum_{j=1}^{n}g_{i,j},$$

where *CS_i_* is the cosine similarity value of the *i*th DEV for the damage, $g_{i}$ is the rate of RED of the *i*th DEV for the damage, $g_{i,j}$ is its *j*th element, and *r_i_* is the REA for the *i*th DEV.

In summary, from a preliminarily updated FE model, the results of the modal analysis conducted according to damage scenarios from the design of experiments for the locations and severities of damage were accumulated as a (DEM). Further, in the recognition process, the rate of change of natural frequencies from operational modal analysis (OMA) was normalized and used as an NWI. When the index exceeds a threshold, it is recognized that damage occurs, and in this state, the DRV was calculated. Furthermore, to identify the damage, the cosine similarity between the DEM and DRV was estimated, the most similar damage scenarios out of the DEM were determined, and they were expressed as the similarity ranking.

### 2.2. Gageocho Ocean Research Station (ORS)

**Figure 4:**
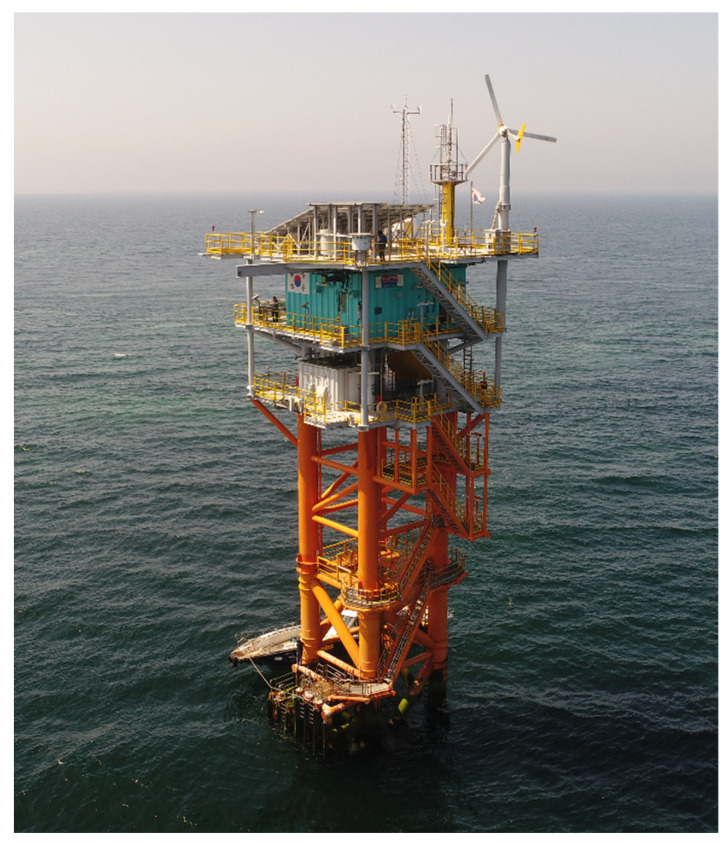
Gageocho ORS [14].

The Gageocho ORS is a jacket-type offshore structure built on a reef called Gageocho in the southwest of the Yellow Sea of South Korea, as shown in Figure 4. The substructure consists of four legs with horizontal and diagonal braces, and there are more than 100 major members. Thirty-six joints are connected to the members. 

Since the ORS was first installed on-site in 2007–2009, as shown in Figure 2, certain events have occurred, and studies related to these events have been conducted. Table 1 presents recent studies and their contributions. Because it was partially destroyed by two strong typhoons, Muipa in 2010 and Kompasu in 2011, a field inspection was performed to diagnose the damage and to investigate its cause. Subsequently, a recovery plan was established and included solutions such as increasing the height of the platform, changing leg member sections, and inserting inner steel piles and concrete grouting in the legs. Based on this, the structural and installation design for rehabilitation was subsequently performed [13]. In 2014, construction was undertaken to rehabilitate the Gageocho ORS, and sensors were installed at that time. Further, in 2017, the dynamic properties of the ORS were analyzed by employing OMA using the measured data. In addition, its dynamic properties up to the fifth mode were analyzed using OMA based on the measured sensor data [28,29]. Subsequently, an FE model was created and modified using the mass reallocation method to match it well with the identified natural frequencies. Consequently, the error rate of its natural frequencies for the measured natural frequencies was found to be less than 1 percent for all five modes [12]. In addition, the design optimization of the jacket structure was performed to obtain a safe and lightweight design exclusive to the concrete grouting that the legs were filled with during retrofitting [30]. These studies addressed sub-topics within the pre-processing of this method, which are represented by the unshaded parts of Figure 3. Thus, a comprehensive estimation of the applicability of the workflow proposed by [27] is still unknown. Therefore, this study provides a practical direction for the application of the cosine similarity-based SHM method.

Thus, this study was conducted based on these preliminary studies. In other words, the FE model updated by [12] was used as the undamaged FE model to generate the DEM; thus, *f_j_* in Equation (4) became the *j*th natural frequency of the updated FE model. The natural frequencies and other necessary information regarding the model in [12] are presented in Table 2.

### 2.3. Damage Scenarios

Several methods have been used to implement damage in FE models. Certain researchers have directly reduced the stiffness parameters of the damaged elements [30], whereas others have modified the cross-section [31,32]. At other times, different approaches have been applied, such as changing boundary conditions [33]. In other words, various methods can be applied to realize model damage in simulations depending on the circumstances.

**Figure 5:**
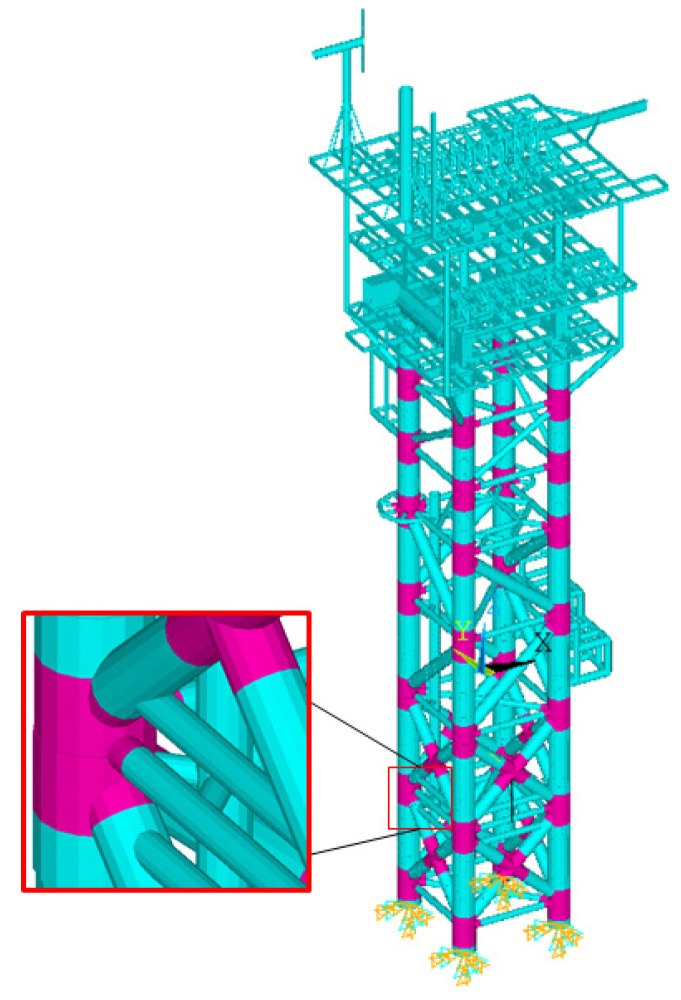
Joints of Gageocho ORS.

Corrosion fatigue is the most common type of damage that affects offshore structures [34,35,36], with joints being the origin of such damage [37]. Therefore, the damage to the joints was addressed in this study. The total number of joints in the jacket structure of the Gageocho ORS is 36, as shown in Figure 5. There was a recent study wherein damage to the lattice structure of the joints was modeled in a manner that reduced the elasticity modulus as well as the elements attached to them [38]. Hence, in this study, damage was also considered as a decrease in the modulus of elasticity as well as in the elements included in a joint, and the damage level is defined by Equation (11).

(11) $$Damage\;level=100\times \frac{E_{DS}-E_{IS}}{E_{IS}}\;\left(\%\right),$$

where *E_DS_* is the elastic modulus of any damaged segment, and *E_IS_* is the elastic modulus of the segment in the intact state. The practical utilization of the approach on an on-site structure requires a precise method for determining the damage level. This is because it is more suitable to match the damage level to the structural capacity or remaining fatigue lifetime in the environment.

The damage scenarios were planned to establish the DEM. The damage scenarios included single as well as multiple damage types, up to three, at the same time. The damage was assumed to be discretized to three damage levels, −15, −30, and −45% in the damage scenarios. Because there are 36 joints, there are 108 single-damage scenarios, that is, 36 joints with three damage levels. For multi-damage, first, 2 or 3 out of the 36 joints were selected. There were 630 and 7140 possible combinations, respectively. Second, each joint from one combination can exhibit one of the three damage levels; that is, nine combinations for two damage types and 27 combinations for three damage types are possible. Therefore, the number of damage scenarios for two and three damage types is 5670 (=630 times 9) and 192,780 (=7140 times 27), respectively. Consequently, the total number of damage scenarios was 198,558.

### 2.4. Assessment on the Change of Natural Frequencies by Damage and Environments

**Figure 6:**
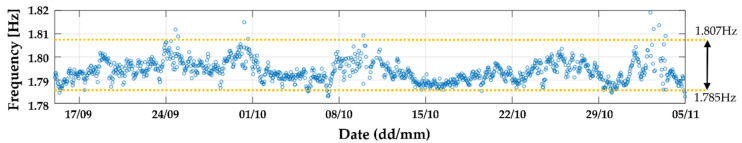
Fluctuation of the first natural frequency of Gageocho ORS [29].

The natural frequencies of a structure vary depending on complex external factors, such as environmental variations and structural damage. This was also observed in the Gageocho ORS, as shown in Figure 6 [29], which displays the fluctuations in the first natural frequency calculated from the measured data. The upper and lower bounds were approximately 1.807 and 1.785 Hz, respectively [29]. Meanwhile, the baseline model exhibited the first natural frequency of 1.807 Hz, as shown in Table 2; therefore, the damage cannot lessen the natural frequency to the lower bound of the fluctuation and might not be easy to determine. The reduction rate to the lower bound of 1.785 Hz for the corresponding intact natural frequency of 1.807 Hz was approximately −0.012175, which is (1.785 – 1.807)/1.807, as shown in Equation (1). The number of damage scenarios wherein the elements of the DEM are less than −0.012175 is 8510 out of 198,558. Specifically, 44 double and 8466 triple damage scenarios induced a reduction in natural frequencies exceeding −0.012175, while there was no single damage scenario among them.

This implies that the change in the dynamic properties arising from the environment is never less than that caused by the damage to a joint because approximately half of the loss of the elastic modulus at any joint cannot trigger changes greater than the maximum change caused by the environment. In other words, accurately identifying the damage with the actual measured natural frequency may be challenging in situations where the environmental influences and results of small damage scenarios are mixed. Further studies are required to address this issue. There are two possible approaches to this. The former involves the creation of the DEM according to the DOE by considering such external changes and not only damage. However, it hinders the determination of the type of environmental factors and the manner in which they quantitatively affect all aspects. Further, they cannot be properly modeled based on an FE model. In addition, the number of damage scenarios would significantly increase due to increasing factors in the DOE, and thus, the computation cost would be much higher. The latter eliminates the influence of external causes on the measured natural frequencies. This appears to be more reasonable than that in general, because, first, the various ocean environmental data that were measured at the Gageocho ORS over a long period of time, such as wind speed and direction, tide, and wave height and period, are available. Second, rapidly growing techniques in data science, such as machine learning, may be an effective solution to remove the environmental impact from actual natural frequencies based on such vast environmental data. Third, if it succeeds in extracting the structural natural frequencies independent of the environmental conditions, their statistical features and the NWI in Equation (6) could be precisely evaluated, thereby helping damage recognition be more evident. This is outside the scope of the current study but will be soon dealt with in depth.

### 2.5. Test Cases for Verification and Damage Reflection Vectors (DRV)

In this study, three test cases, A, B, and C, were tested. Test cases A and B included three damage states with 7, 15, and 31 damaged joints, as shown in Table 3 and Table 4. Damage state 1 is single damage on joint 7; damage state 2 is double damage on joints 7 and 15; and damage state 3 is triple damage on joints 7, 15, and 31, respectively. The damage levels of test case A (−14, −29, and −44%) were very close to the damage levels of −15, −30, and −45% used for the DEM. In contrast, the damage levels of test case B, −11%, −25, and −49% were not close to the damage levels of the DEM. Finally, test case C only addressed the single damage at damaged joint 7, but the 11 damage states according to the 11 damage levels ranging from −25 to −35% were compared to one another as shown in Table 5. Table 6 presents the natural frequencies of the ORS with the damaged states and the DRVs for the cosine similarity comparison.

## 3. Results and Discussion

### 3.1. Test Case A

Table 7 lists the results for test case A. The first row shows the actual damage, and the others are the damage scenarios that were ranked in the top five by the proposed method. With respect to damage state A1, the value of the cosine similarity of the top-ranked damage scenario was 0.999945, and a damaged joint 7 and a damage level of −30% were predicted. Regarding damage state A2, the top-ranked damage scenario included the two damages at joint 7, with a damage level of −30%, and at joint 15 with a damage level of −15%. Thus, the first-ranked scenario of damage state A3 detected triple damage at joints 7, 15, and 31. In addition, the damage levels of these joints were predicted to be −30, −15, and −45%, respectively. Consequently, the damage locations as well as the damage levels of all the damage states in test case A were precisely predicted.

### 3.2. Test Case B

Table 8 lists the results for test case B. The first row is the actual damage, whereas the others are the damage scenarios that were ranked in the top five by the proposed method. Compared to the single damage of damage state B1, the top-ranked scenario provided triple damage at joints 3, 5, and 15, with a cosine similarity of 0.999873. Thus, the proposed method was unable to correctly diagnose the damage that occurred. Among the top five ranked scenarios, only the top second scenario included the damaged joint 7, but it also predicted triple damage, which is not the same as single damage. Further, regarding damage state B2, which is among the double damage scenarios, the proposed method yielded the damage scenario 38,109 as the most similar scenario, which is triple damage at joints 3, 5, and 15. Although the damage at joint 15 was found, the damage level was not accurately determined, and another damage location, that is, joint 7, was not detected in the top five scenarios.

In contrast, regarding damage state B3, the damaged joints in the top-ranked scenario were well matched to the actual damaged joints, 7, 15, and 31. The predicted damage level of −30% at joint 7 was quite similar to the actual damage level of −25% at that joint; however, the predicted damage level of −45% at joint 15 was significantly overestimated compared to the actual damage level of −11%. Moreover, the predicted damage level of −30% at joint 31 was underestimated compared to the actual damage level of −49% at that joint.

Contrary to the results of test case A, the proposed method was not able to accurately detect the location of and how severe the damage in test case B would be. This is because the damage levels of test case A, that is, −14, −29, and −44%, are very close to the discretized damage levels of the damage scenarios, −15, −30, and −45%, whereas the damage levels of test case B, that is, −11, −25, and −49%, were not. Consequently, it can be concluded that the proposed method based on the cosine similarity between the vectors of the change rate in the natural frequencies can only provide the correct answer when the damage state is close to a damage scenario.

### 3.3. Test Case C

**Figure 7:**
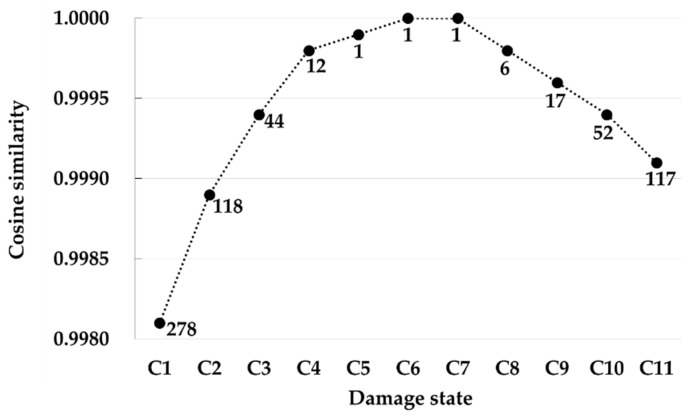
Cosine similarity and ranking of damage scenario C for the eleven damage states (C1–C11) of damage case C.

As shown in Table 5, test case C exhibited 11 single damage scenarios at joint 7, with the damage level ranging from −25 to −35% at an interval of 1%. Meanwhile, there were three single damage scenarios at joint 7 in the DEM to calculate the cosine similarity, with their damage levels being −15, −30, and −45%. Therefore, the damage states are considered to be closest to the damage scenario with a damage level of −30% at joint 7, which is, hereinafter, referred to as damage scenario C. This is because, first, they have the same damaged joint 7, and second, the damage levels of damage scenario C and the 11 damage states of test case C exhibit the least amount difference within ±5%. Figure 7 represents the ranking and values of the cosine similarity of damage scenario C for the 11 damage states. Consequently, the proposed method concluded that damage scenario C was the top-ranked in the case of only three damage states, C5, C6, and C7, among the 11 damage states. The damage level of these damage states was observed to be extremely close to the damage level of damage scenario C within ±1%. Therefore, the proposed method enables the detection of the location of and the manner in which serious damage occurred in the case where a certain damage state was almost identical to a damage scenario in the DEM; otherwise, it is restricted to accurately detecting damage, particularly in a complicated lattice structure, such as in this jacket.

### 3.4. Comprehensive Discussion

The variation in the natural frequencies due to the environment, as shown in Figure 6, overwhelms the change range of the natural frequencies due to damage. In addition, the cosine similarity-based on the damage detection method is only capable of diagnosing single and multiple damage scenarios if their damage levels are very close to being within 1% of the damage levels of the damage scenarios. Thus, to overcome this obstacle, first, the relationship between the dynamic characteristics of the structure and the environment, such as the temperature and tidal level, should be clarified accurately such that the error is much less than 1%. This is to correct the uncertainty of the natural frequencies. Second, the damage scenarios must be more thoroughly designed with a much finer interval, that is, approximately 3% of the damage level. However, this approach would significantly increase the calculation cost to construct the DEM, which would render the computation of all damage scenarios nearly impossible. Hence, effective techniques to establish a database, such as the metamodeling in Figure 3, are required.

Therefore, this technique can be extended to offshore wind turbine systems in practice. In addition to the environmental effects mentioned above, the dynamic features of offshore wind turbine systems must be considered in future research. Various studies have been published on offshore wind turbine systems. As representative instances, certain sequential studies have been performed for spar-type offshore wind turbine systems [39,40,41]. First, the natural frequency of a wind turbine tower with end-mass components was investigated [39]. Second, the dynamic behavior of the 5 MW wind turbine was analyzed in ocean environments, such as in current, wind, and wave conditions [40]. Finally, the fatigue lifetime of the roller bearing was studied under environmental excitation [41]. Similar to the series of studies, the characteristics of a specific offshore wind turbine system to which this SHM method is applied, for example, the dynamic properties, responses, and fatigue life, may be studied and then utilized in the SHM method in the future.

## 4. Conclusions

Based on the most critical issue of the present time, global warming, this study premises the urgency of sustainable energy development in the offshore field and the uncertain safety of structures due to abnormal climate change in the future. Furthermore, this study highlighted that traditional maintenance procedures were not suited to treat these safety issues, and instead, a more immediate and easily interpretable method was needed to identify the structural state. Consequently, as an alternative, this study investigated whether cosine similarity-based damage identification could be effectively applied to an actual offshore structure.

For the jacket-type substructure of the Gageocho Ocean Research Station, various damage states, that is, single, double, and triple damage scenarios, with damage levels close to or far from the damage level of the comparison targets, were tested. Consequently, the cosine similarity-based damage detection technique only enabled the accurate determination of damage in cases where the damage state was quite close to a damage scenario within the range of the ±1% damage level.

Thus, the damage levels must be discretized to be much finer (i.e., almost 3% interval) to guarantee the accuracy of the method. However, this causes an increase in the computation costs for constructing the damage scenarios and the damage estimation matrix; thus, calculating every damage scenario is nearly impossible. Therefore, an efficient approach to reduce computing time, such as metamodeling, is required.

In addition, the change in the natural frequencies induced by the damage was found to be much smaller than that triggered by the environmental variation. Hence, fairly precise measurements and processing to extract the natural frequencies with a high level of accuracy are essential to eliminating the impact of the environment on the fluctuations in the natural frequencies, or the environmental effects should be embraced in the establishment of the damage estimation matrix. Therefore, research is needed to clarify which environmental factors result in what degree of change in the natural frequencies.

## Figures and Tables

**Table 1:** Summary of recent research studies relevant with this topic.

Reference	Published Year	Contribution	Part in Figure 3
[13]	2012	Damage cause analysis; Rehabilitation design	Structure design
[28]	2017	Short-term operational modal analysis	Operational Modal Analysis
[29]	2019	Long-term operational modal analysis	Operational Modal Analysis
[12]	2020	Simulation model updating	FE Model Update
[30]	2021	Lightweight structural design optimization	Structure design
[27]	2019	Proposal of the cosine similarity-based SHM	

**Table 2:** Elastic modulus of steel (*E_IS_*) and natural frequencies (*f_j_*) of the updated Gageocho FE model.

*E_IS_*	*f_1_*	*f_2_*	*f_3_*	*f_4_*	*f_5_*
215 GPa	1.807 Hz	1.809 Hz	2.654 Hz	5.586 Hz	5.685 Hz

**Table 3:** Test case A.

State	Damaged Joint	Damage Level
A1	7	−29%
A2	7	−29%
	15	−14%
A3	7	−29%
	15	−14%
	31	−44%

**Table 4:** Test case B.

State	Damaged Joint	Damage Level
B1	7	−25%
B2	7	−25%
	15	−11%
B3	7	−25%
	15	−11%
	31	−49%

**Table 5:** Test case C.

State	Damaged Joint	Damage Level
C1	7	−25%
C2		−26%
C3		−27%
C4		−28%
C5		−29%
C6		−30%
C7		−31%
C8		−32%
C9		−33%
C10		−34%
C11		−35%

**Table 6:** Natural frequencies and damage reflection vector (DRV) of test cases A, B, and C.

State	Natural Frequencies [Hz]					DRV				
	1	2	3	4	5	1	2	3	4	5
A1	1.8035	1.8076	2.6528	5.5770	5.6780	−0.0019	−0.0008	−0.0004	−0.0016	−0.0012
A2	1.8023	1.8073	2.6521	5.5745	5.6778	−0.0026	−0.0009	−0.0007	−0.0021	−0.0013
A3	1.8014	1.8068	2.6508	5.5545	5.6761	−0.0031	−0.0012	−0.0012	−0.0056	−0.0016
B1	1.8044	1.8077	2.6530	5.5787	5.6793	−0.0015	−0.0007	−0.0004	−0.0013	−0.0010
B2	1.8034	1.8075	2.6524	5.5768	5.6791	−0.0020	−0.0008	−0.0006	−0.0016	−0.0010
B3	1.8023	1.8070	2.6509	5.5526	5.6770	−0.0026	−0.0011	−0.0012	−0.0060	−0.0014
C1	1.8021	1.8074	2.6525	5.5741	5.6758	−0.0027	−0.0009	−0.0005	−0.0021	−0.0016
C2	1.8024	1.8075	2.6526	5.5746	5.6762	−0.0026	−0.0009	−0.0005	−0.0020	−0.0015
C3	1.8026	1.8075	2.6526	5.5751	5.6766	−0.0024	−0.0008	−0.0005	−0.0019	−0.0015
C4	1.8029	1.8075	2.6527	5.5756	5.6770	−0.0023	−0.0008	−0.0005	−0.0019	−0.0014
C5	1.8031	1.8075	2.6527	5.5761	5.6773	−0.0022	−0.0008	−0.0005	−0.0018	−0.0013
C6	1.8033	1.8076	2.6528	5.5765	5.6777	−0.0020	−0.0008	−0.0005	−0.0017	−0.0013
C7	1.8035	1.8076	2.6528	5.5770	5.6780	−0.0019	−0.0008	−0.0004	−0.0016	−0.0012
C8	1.8038	1.8076	2.6529	5.5774	5.6784	−0.0018	−0.0008	−0.0004	−0.0015	−0.0012
C9	1.8040	1.8077	2.6529	5.5778	5.6787	−0.0017	−0.0007	−0.0004	−0.0015	−0.0011
C10	1.8042	1.8077	2.6530	5.5783	5.6790	−0.0016	−0.0007	−0.0004	−0.0014	−0.0011
C11	1.8044	1.8077	2.6530	5.5787	5.6793	−0.0015	−0.0007	−0.0004	−0.0013	−0.0010

**Table 7:** Damage scenarios ranked in the top five and the corresponding cosine similarities (CS) of test case A.

Ranking	A1				A2				A3			
	CS	Scenario	Joint	Level	CS	Scenario	Joint	Level	CS	Scenario	Joint	Level
-	-	-	7	−29%	-	-	7 15	−29% −14%	-		7	−29%
											15	−14%
											31	−44%
1	0.999945	20	7	−30%	0. 999962	1930	7 15	−30% −15%	0.999984	94080	7	−30%
											15	−15%
											31	−45%
2	0.999940	26915	2	−45%	0. 999865	67135	5	−30%	0.999753	99887	7	−30%
			9	−30%			9	−30%			35	−30%
			14	−30%			15	−15%			36	−30%
3	0.999887	28967	2	−45%	0. 999832	95167	7	−45%	0.999743	99617	7	−30%
			12	−30%			17	−15%			31	−30%
			15	−30%			32	−15%			36	−30%
4	0.999797	41171	3	−45%	0. 999797	95275	7	−45%	0.999728	93513	7	−30%
			9	−30%			17	−15%			14	−15%
			14	−30%			36	−15%			31	−45%
5	0.999729	92953	7	−45%	0. 999762	52588	4	−45%	0.999718	71481	5	−30%
			13	−15%			6	−15%			15	−15%
			32	−15%			25	−15%			35	−45%

**Table 8:** Damage scenarios ranked in the top five and the corresponding cosine similarities (CS) of test case B.

Ranking	B1				B2				B3			
	CS	Scenario	Joint	Level	CS	Scenario	Joint	Level	CS	Scenario	Joint	Level
-	-	-	7	−25%	-	-	7 15	−25% −11%	-		7	−25%
											15	−11%
											31	−49%
1	0.999873	38121	3 5 15	−45% −30% −45%	0.999979	38109	3 5 15	−30% −15% −45%	0.999898	99620	7	−30%
											15	−45%
											31	−30%
2	0.999826	10471	1	−45%	0.999897	70831	5	−30%	0.999881	77925	6	−15%
			7	−30%			14	−15%			7	−15%
			21	−15%			32	−15%			35	−45%
3	0.999815	37941	3	−15%	0.999891	23335	2	−15%	0.999881	88737	6	−30%
			5	−30%			4	−45%			32	−30%
			9	−45%			27	−15%			35	−45%
4	0.999815	23685	2	−15%	0.999869	37591	3	−15%	0.999861	99701	7	−30%
			5	−30%			4	−45%			32	−45%
			9	−45%			27	−15%			35	−30%
5	0.999802	11691	1	−45%	0.999857	66994	5	−15%	0.999842	99696	7	−30%
			9	−45%			9	−45%			32	−15%
			11	−45%			10	−15%			35	−45%
